# Non-sequential double ionization with near-single cycle laser pulses

**DOI:** 10.1038/s41598-017-07635-5

**Published:** 2017-08-08

**Authors:** A. Chen, M. Kübel, B. Bergues, M. F. Kling, A. Emmanouilidou

**Affiliations:** 1 Department of Physics and Astronomy, University College London, Gower Street, London, WC1E 6BT United Kingdom; 2 Joint Laboratory for Attosecond Science, University of Ottawa and National Research Council, 100 Sussex Drive, Ottawa, Ontario K1A 0R6 Canada; 3 Max-Planck-Institut für Quantenoptik, D-85748 Garching, Germany; 4 Department für Physik, Ludwig-Maximilians-Universität, D-85748 Garching, Germany

## Abstract

A three-dimensional semiclassical model is used to study double ionization of Ar when driven by a near-infrared and near-single-cycle laser pulse for intensities ranging from 0.85 × 10^14^ W/cm^2^ to 5 × 10^14^ W/cm^2^. Asymmetry parameters, distributions of the sum of the two electron momentum components along the direction of the polarization of the laser field and correlated electron momenta are computed as a function of the intensity and of the carrier envelope phase. A very good agreement is found with recently obtained results in kinematically complete experiments employing near-single-cycle laser pulses. Moreover, the contribution of the direct and delayed pathways of double ionization is investigated for the above observables. Finally, an experimentally obtained anti-correlation momentum pattern at higher intensities is reproduced with the three-dimensional semiclassical model and shown to be due to a transition from strong to soft recollisions with increasing intensity.

## Introduction

Non-sequential double ionization (NSDI) in intense near-infrared laser fields is a fundamental process with electron-electron correlation playing a key role^1–3^. Considerable information regarding NSDI has been obtained from kinematically complete experiments, i.e., the momenta of the escaping electrons and ions are measured in coincidence^4^. Most of these experiments employ multi-cycle laser pulses allowing for multiple recollisions to occur before both electrons ionize. Multiple recollisions complicate the electron dynamics and render the comparison with theory difficult. Recently, however, kinematically complete experiments succeeded in confining NSDI to a single laser cycle by using carrier-envelope phase (CEP)-controlled few- and near-single-cycle pulses^5, 6^. These experiments with near-single-cycle pulses allow for an easier comparison between theory and experiment.

To interpret the double ionization spectra of driven Ar measured using near-single-cycle laser pulses, a simple one-dimensional (1D) classical model was put forth^6–8^. This model relies on the assumption that the dominant pathways of double ionization are, for small and intermediate intensities, delayed non-sequential ionization and, for higher intensities, sequential ionization. For strongly-driven Ar, intermediate intensities refer to the intensity range from 2 × 10^14^ W/cm^2^ to 4 × 10^14^ W/cm^2^. This model neglects the contribution of another major pathway of double ionization, namely, direct ionization as well as the Coulomb potential. This 1D model did not achieve a quantitative agreement with the complete set of available experimental data over the whole intensity range. Delayed ionization—also referred to as recollision-induced excitation with subsequent field ionization, RESI^9, 10^, and direct ionization are two main pathways of NSDI. An interesting finding of these near-single cycle experiments was that the correlated momenta components of the two escaping electrons along the direction of the laser field have a cross-shaped pattern for an intensity around 10^14^ W/cm^2^ ^6–8^. A cross-shaped correlated electron momenta pattern due to the delayed double ionization mechanism was previously identified in the context of strongly-driven He at an intensity of 9 × 10^14^ W/cm^2^ and for a wavelength of 400 nm^11^. In a cross-shaped correlated electron momenta pattern the double ionization probability is the highest when the component of the momentum along the direction of the laser field is very small for one electron while it takes a wide range of values for the other electron, see the experimental correlated electron momenta at 10^14^ W/cm^2^ in Fig. 5. In the context of strongly-driven Ar, the above described 1D model attributed the cross-shaped pattern of the correlated electron momenta to the delayed pathway of double ionization^7, 8^. A quantum mechanical calculation, which neglects the Coulomb potential, was used to refine the contribution of the delayed pathway of double ionization to the cross-shaped correlated electron momenta pattern^12^. This calculation identified the key role that the symmetry of the excited state plays in the final shape of the correlated momenta.

In this work, using a three-dimensional (3D) semiclassical model, NSDI of Ar is studied when Ar is driven by 750 nm near-single-cycle laser pulses at intensities ranging from 0.85 × 10^14^ W/cm^2^ to 5 × 10^14^ W/cm^2^. In this 3D model the only approximation is in the initial state. There is no approximation during the time propagation. That is, all Coulomb forces and the interaction of each electron with the laser field are fully accounted for. Moreover, when analyzing the numerically obtained doubly-ionized events, no assumptions are made regarding the prevailing mechanism of double ionization and we use no free parameter. This is not the case for the 1D model^8^. In addition, the Coulomb singularity is fully accounted for using regularized coordinates^13^. This is an advantage over models which soften the Coulomb potential^14^. Previous successes of this 3D model include identifying the mechanism responsible for the fingerlike structure in the correlated electron momenta^15^, which was predicted theoretically^16^ and was observed experimentally for He driven by 800 nm laser fields^17, 18^. Moreover, this model was used to investigate direct versus delayed pathways of NSDI for He driven by a 400 nm laser field while achieving excellent agreement with fully ab-initio quantum mechanical calculations^19^. Using this model, in this work, several observables are computed for different intensities of strongly-driven Ar. These observables are the sum of the two electron momentum components along the direction of the polarization of the laser field and the double differential probability of the two electron momentum components along the polarization direction of the laser field, i.e. the correlated electron momenta. Furthermore, the amplitude and the phase of the asymmetry parameter that determines the difference of the ions escaping with positive versus negative momentum along the polarization direction of the laser field are computed as a function of the carrier envelope phase (CEP) and the intensity.

Previously obtained experimental results over the whole intensity range^6–8^ are in better agreement with the computed results obtained using the 3D semiclassical model rather than with the computed results obtained with the 1D model in ref. 8. Throughout this work the computed results are compared with the experimental results that were recently published in and adopted from ref. 8 where the data acquisition and analysis is described in detail. Briefly, CEP stable laser pulses with a full-width-half-maximum pulse duration of 4 fs and a center wavelength of 750 nm are focused onto a cold-gas jet of argon atoms inside a reaction microscope. There, the momenta of ions and electrons generated in the laser focus via strong field ionization are recorded in coincidence as a function of the intensity and of the CEP of the laser pulse. The CEP is measured with a precision of roughly 200 mrad. Motivated by the good agreement we find in this work between theory and experiment, the strength of the 3D semiclassical model in fully accounting for the electron dynamics is utilized to identify the prevailing pathway of double ionization as a function of intensity. In addition, for a small intensity around 10^14^ W/cm^2^, the dependence of the double ionization pathways on CEP is computed using the 3D semiclassical model. Finally, the transition from strong to soft recollisions is identified as the main reason for the experimentally observed escape of the two electrons with opposite momenta at higher intensities^20^.

## Method

For the current studies, a 3D semiclassical model is employed that is formulated in the framework of the dipole approximation^15^. The initial state in the 3D model entails one electron tunneling through the field-lowered Coulomb potential with the Ammosov-Delone-Krainov (ADK) formula^21, 22^. To obtain the tunnel ionization rate for Ar, in the ADK formula the first ionization energy of Ar, i.e. ${{\rm{I}}}_{{{\rm{p}}}_{{\rm{1}}}}={\rm{0.579}}\,{\rm{a}}{\rm{.u}}{\rm{.}}$ and the effective charge Z = 1 are used. The exit point of the tunnel-ionized electron is along the direction of the laser field and is computed using parabolic coordinates^23^. The momentum along the direction of the electric field is zero while the transverse one is given by a Gaussian distribution^21, 22^. Thus, the formulation of the initial state of the electron that tunnel-ionizes is a quantum mechanical one. The rest of the formulation in the 3D model, i.e the initial state of the remaining electron and the time propagation of both electrons, is classical. The remaining electron is initially described by a microcanonical distribution^24^. In what follows, the initially tunneling and bound electrons are denoted as electrons 1 and 2, respectively. The weight of each classical trajectory i that we propagate in time is given by

1 $${{\rm{W}}}_{{\rm{i}}}={{\rm{W}}}_{{\rm{i}}}^{{\rm{1}}}\cdot {{\rm{W}}}_{{\rm{i}}}^{{\rm{2}}},$$

where

2 $${{\rm{W}}}_{{\rm{i}}}^{{\rm{1}}}\propto {(\frac{1}{| \vec{{\rm{E}}}({{\rm{t}}}_{{\rm{0}}})| })}^{{{\rm{2n}}}^{* }-{\rm{1}}}\,exp\,(-\frac{2{\kappa }^{3}}{3| \vec{{\rm{E}}}({{\rm{t}}}_{{\rm{0}}})| })$$

is the ADK ionization rate^21, 22^ at the time t_0_ of tunnel-ionization. n^*^ is the effective principal quantum number given by ${{\rm{I}}}_{{{\rm{p}}}_{{\rm{1}}}}={{\rm{Z}}}^{{\rm{2}}}/{{\rm{2n}}}^{* 2}$ and $\kappa =\sqrt{2{{\rm{I}}}_{{{\rm{p}}}_{{\rm{1}}}}}$. ${{\rm{W}}}_{{\rm{i}}}^{{\rm{2}}}$ is the weight for electron 1 to have a transverse velocity equal to ${{\rm{v}}}_{\perp }$ at the time t_0_:

3 $${{\rm{W}}}_{{\rm{i}}}^{{\rm{2}}}\propto \frac{{{\rm{v}}}_{\perp }}{| \vec{{\rm{E}}}({{\rm{t}}}_{{\rm{0}}})| }\,exp\,(-\frac{{{\rm{v}}}_{\perp }^{{\rm{2}}}\kappa }{| \vec{{\rm{E}}}({{\rm{t}}}_{{\rm{0}}})| }).$$

t_0_ is the time electron 1 tunnel-ionizes through the field-lowered Coulomb potential. In our computation, for each classical trajectory, the tunnel-ionization time is selected randomly in the time interval that the laser field is on. In this work the laser field is linearly polarized and is given by

4 $$\vec{{\rm{E}}}(t)={{\rm{E}}}_{{\rm{0}}}{{\rm{e}}}^{(-{\rm{2ln2}}{(\frac{{\rm{t}}}{\tau })}^{{\rm{2}}})}\,cos\,(\omega {\rm{t}}+\varphi )\,\hat{{\rm{z}}},$$

where *τ* = 4 fs is the full-width-half-maximum pulse duration, *ω* = 0.061 a.u. (750 nm) is the frequency, E_0_ is the strength and *ϕ* is the CEP of the laser field. For this laser field the tunnel-ionization time is selected randomly effectively in the time interval (−2*τ*, 2*τ*). Once the initial conditions for each electron are specified, the position and momentum of each electron are propagated forward in time, starting at time t_0_, by solving the classical equations of motion. The time propagation is determined by the three-body Hamiltonian of the two electrons with the nucleus kept fixed. All Coulomb forces are accounted for: the interaction of each electron with the nucleus and the laser field and the electron-electron interaction are all included in the time propagation. During the time propagation each electron is interacting with the nucleus with charge Z = 2. A trajectory is labeled as a doubly-ionized event if asymptotically, i.e. t → ∞, the energies of both electrons are positive. The double and single ionization probabilities are given by

5 $${{\rm{P}}}_{{\rm{DI}}}=\frac{{\sum }_{{\rm{i}}}^{{{\rm{N}}}_{{\rm{DI}}}}{{\rm{W}}}_{{\rm{i}}}}{{\sum }_{{\rm{i}}}^{{\rm{N}}}{{\rm{W}}}_{{\rm{i}}}},\quad {{\rm{P}}}_{{\rm{SI}}}=\frac{{\sum }_{{\rm{i}}}^{{{\rm{N}}}_{{\rm{SI}}}}{{\rm{W}}}_{{\rm{i}}}}{{\sum }_{{\rm{i}}}^{{\rm{N}}}{{\rm{W}}}_{{\rm{i}}}}$$

where N_DI_, N_SI_ and N are the numbers of doubly-ionized, singly-ionized and all events, respectively.

For the results presented in this work, the intensities considered range from 0.85 × 10^14^ W/cm^2^ to 5 × 10^14^ W/cm^2^. At 0.85 × 10^14^ W/cm^2^, 12 CEPs are considered ranging from *ϕ* = 15° to *ϕ* = 345° in steps of 30°; for each *ϕ*, 1.5 × 10^4^ doubly-ionized events are obtained as a result of running 400, 12-hour jobs; one job corresponds to 1 CPU. For all other intensities, 24 CEPs are considered ranging from *ϕ* = 0° to *ϕ* = 360° in steps of 15°. For each *ϕ*, at 10^14^ W/cm^2^, 1.4 × 10^14^ W/cm^2^, 2 × 10^14^ W/cm^2^, 3 × 10^14^ W/cm^2^, 4 × 10^14^ W/cm^2^ and 5 × 10^14^ W/cm^2^ the doubly-ionized events thus obtained are 1.5 × 10^4^, 5 × 10^4^, 10^5^, 1.8 × 10^5^, 3 × 10^5^ and 6 × 10^5^, respectively as a result of running 100, 12-hour jobs. For the results presented regarding total double ionization the average has been taken over all CEPs for each intensity. From the above, it is clear that the computations required, particularly for the lower intensities, are challenging, since, it is time-consuming to obtain enough doubly-ionized events that render the statistical error very small for each intensity and for each of the 12 or 24 CEPs. The statistical error is proportional to ${\rm{1}}/\sqrt{{{\rm{N}}}_{{\rm{DI}}}}$ ^24^. The intense computations required is the reason results are obtained for seven intensities in the range from 0.85 × 10^14^ W/cm^2^ to 5 × 10^14^ W/cm^2^. Using the results obtained at these seven intensities an average over the focal volume is performed^25^ to directly compare with experiment. It is, however, noted that computations at a larger number of intensities are needed to account more accurately for the focal volume effect (FVE). For the results presented, it is stated explicitly when focal volume averaging is included and when it is not.

## Results

### Double ionization and pathways

**Figure 1:**
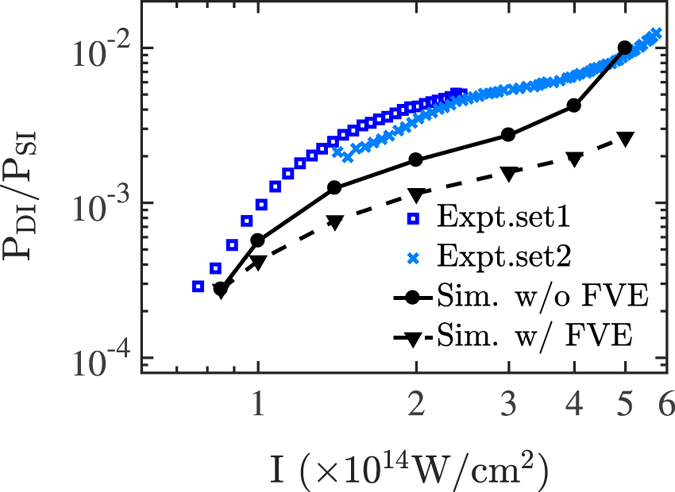
Ratio of double to single ionization probability as a function of intensity. Experimental results^8^ are denoted by dark blue squares and light blue crosses and computed results are presented by a solid line with black circles when the focal volume effect is not accounted for and by a dashed-line with triangles when the focal volume effect is accounted for. The difference in the two experimental sets results from slightly different averaging over the focal volume^8^.

In Fig. 1, the ratio of double to single ionization probability is computed as a function of the laser intensity and compared to the experimental results^8^. It is found that the computed ratio of double to single ionization probability reproduces well the overall pattern of the observed ratio. The computed ratio is found to be at most a factor of two smaller than the observed ratio and by a factor of 3.5 when the focal volume effect is accounted for. This difference possibly suggests that the effective charge of Z = 2 used to model the attractive Coulomb potential in the 3D semiclassical model during time propagation overestimates the Coulomb attraction.

**Figure 2:**
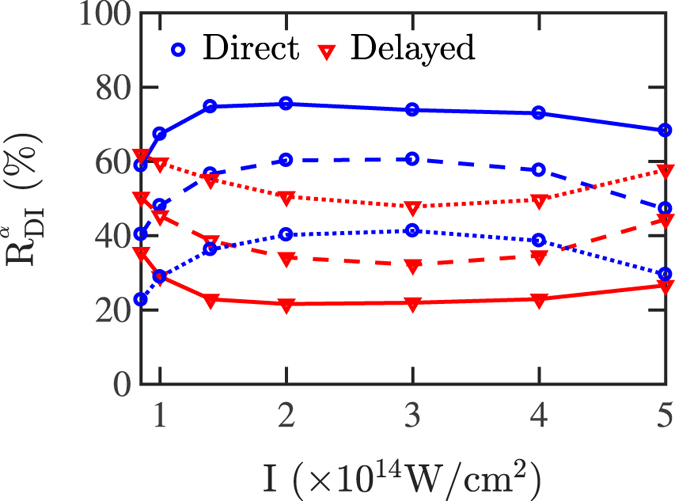
Percentage contribution of the direct (blue circles) and the delayed (red triangles) pathways of double ionization as a function of the laser intensity for t_diff_ = 1/10 T (solid lines), t_diff_ = 1/20 T (dashed lines) and t_diff_ = 1/40 T (dotted lines). The focal volume effect is not accounted for.

Once the doubly-ionized events are obtained using the 3D semiclassical model, an analysis of the classical trajectories is performed in time in order to identify the contribution of the direct and the delayed pathway of NSDI as a function of the laser intensity. The main two double ionization energy transfer pathways are identified by using the time difference between the recollision time t_rec_ and the ionization time of each electron ${{\rm{t}}}_{{\rm{ion}}}^{{\rm{i}}}$, with i = 1, 2, for each doubly-ionized classical trajectory. The recollision time is defined as the time of minimum approach of the two electrons and is identified by the maximum in the electron pair potential energy. The ionization time for each electron is defined as the time when the sum of the electron’s kinetic energy (using the canonical momentum) and the potential energy due to the electron’s interaction with the nucleus becomes positive and remains positive thereafter. The canonical momentum of an electron is given by **p** − **A**, with **A** the vector potential. The ionization time of electron 1 is, thus, not necessarily the time t_0_ this electron tunnel-ionizes at the start of the time propagation. This energy is referred to as compensated energy and was introduced in ref. 26. A doubly-ionized trajectory is labeled as delayed or direct depending on the time differences ${{\rm{t}}}_{{\rm{ion}}}^{{\rm{1}}}-{{\rm{t}}}_{{\rm{rec}}}$ and ${{\rm{t}}}_{{\rm{ion}}}^{{\rm{2}}}-{{\rm{t}}}_{{\rm{rec}}}$. Specifically,

6 $$\begin{array}{l}|{{\rm{t}}}_{{\rm{ion}}}^{{\rm{1}}}-{{\rm{t}}}_{{\rm{rec}}}| < {{\rm{t}}}_{{\rm{diff}}}\,{\&}\,{{\rm{t}}}_{{\rm{ion}}}^{{\rm{2}}} < {{\rm{t}}}_{{\rm{ion}}}^{{\rm{1}}}\quad \quad \quad ({\rm{a}})\\ |{{\rm{t}}}_{{\rm{ion}}}^{{\rm{2}}}-{{\rm{t}}}_{{\rm{rec}}}| < {{\rm{t}}}_{{\rm{diff}}}\,{\&}\,{{\rm{t}}}_{{\rm{ion}}}^{{\rm{1}}} < {{\rm{t}}}_{{\rm{ion}}}^{{\rm{2}}}\quad \quad \quad ({\rm{b}})\end{array}\quad {\rm{Direct}}$$

7 $$\begin{array}{l}{{\rm{t}}}_{{\rm{ion}}}^{{\rm{1}}}-{{\rm{t}}}_{{\rm{rec}}} > {{\rm{t}}}_{{\rm{diff}}}\,{\&}\,{{\rm{t}}}_{{\rm{ion}}}^{{\rm{2}}}-{{\rm{t}}}_{{\rm{rec}}} < {{\rm{t}}}_{{\rm{diff}}}\quad \quad \quad ({\rm{a}})\\ {{\rm{t}}}_{{\rm{ion}}}^{{\rm{2}}}-{{\rm{t}}}_{{\rm{rec}}} > {{\rm{t}}}_{{\rm{diff}}}\,{\&}\,{{\rm{t}}}_{{\rm{ion}}}^{{\rm{1}}}-{{\rm{t}}}_{{\rm{rec}}} < {{\rm{t}}}_{{\rm{diff}}}\quad \quad \quad ({\rm{b}})\end{array}\quad {\rm{Delayed}}$$

8 $${{\rm{t}}}_{{\rm{ion}}}^{{\rm{1}}}-{{\rm{t}}}_{{\rm{rec}}} > {{\rm{t}}}_{{\rm{diff}}}\,{\&}\,{{\rm{t}}}_{{\rm{ion}}}^{{\rm{2}}}-{{\rm{t}}}_{{\rm{rec}}} > {{\rm{t}}}_{{\rm{diff}}}\quad \quad \quad \quad {\rm{Double}}\,{\rm{Delayed}}$$

where t_diff_ is a positive arbitrary parameter. The percentage of doubly-ionized events labeled as delayed or direct, out of all doubly-ionized events, depends on our choice of the time difference t_diff_. These percentages are given by

9 $${{\rm{R}}}_{{\rm{DI}}}^{\alpha }=\frac{{\sum }_{{\rm{i}}}^{{{\rm{N}}}_{{\rm{DI}}}^{\alpha }}{{\rm{W}}}_{{\rm{i}}}}{{\sum }_{{\rm{i}}}^{{{\rm{N}}}_{{\rm{DI}}}}{{\rm{W}}}_{{\rm{i}}}},$$

where ${{\rm{N}}}_{{\rm{DI}}}^{\alpha }$ is the number of *α* labelled doubly-ionized events, with *α* denoting the direct or delayed events. Thus, the probability of doubly-ionized events labeled as delayed or direct, out of all events, is given by ${{\rm{P}}}_{{\rm{DI}}}^{\alpha }={{\rm{R}}}_{{\rm{DI}}}^{\alpha }{{\rm{P}}}_{{\rm{DI}}}$. t_diff_ should not be chosen neither very large, such as 1/4 T, or very small such as 1/40 T. Choices in between are reasonable and lead to similar trends of the two prevailing pathways of double ionization. This is shown in Fig. 2 where the percentages of direct and delayed doubly-ionized events are plotted for t_diff_ equal to 1/10 T, 1/20 T and 1/40 T as a function of the intensity of the laser field. It is found that the contribution of the direct and the delayed pathways to double ionization as a function of intensity displays general trends that do not significantly depend on the choice of t_diff_. Both the direct and the delayed pathways of double ionization significantly contribute at all intensities. Thus, the direct pathway can not be neglected as was done in previous models. The direct pathway contributes the most for intermediate intensities. In Fig. 2, at a high intensity above 4 × 10^14^ W/cm^2^, it is shown that the contribution of the direct pathway of double ionization starts decreasing. At this high intensity a transition from strong to soft recollisions takes place, as discussed in the following. It is found that double delayed events contribute no more than 15% for the smallest intensity even when the time difference is chosen small and equal to 1/40 T. t_diff_ = 1/10 T is chosen for the results presented in this work. We find that with this choice of t_diff_ the distributions of the sum of the two electron momentum components along the polarization direction of the laser field for the direct and the delayed pathways of double ionization are the closest to what is expected from ref. 10. That is, the former distribution dips while the latter one peaks around zero.

We find that different results are obtained if instead of the compensated energy the energy of each electron is used to identify the ionization time. Namely, one finds that at an intensity of 0.85 × 10^14^ W/cm^2^ almost all classical trajectories are identified as double delayed. This was the conclusion in ref. 5. Using the actual energy to identify the ionization time at an intensity of 3 × 10^14^ W/cm^2^ results in the direct pathway of double ionization still only contributing 20%. However, this is not a reasonable result. At 3 × 10^14^ W/cm^2^ 3.17 U_p_ is equal to 50 eV which is much higher than the second ionization energy of Ar. Moreover, the recollision at this intensity is strong, which is discussed in the section for the correlated electron momenta as a function of intensity, and so the direct pathway of double ionization should contribute significantly. Thus, the compensated energy is employed to identify the ionization time in this work which leads to both the direct and delayed pathway being the main pathways of double ionization in agreement with ref. 14 for the smallest intensity.

### Distribution of the sum of the electron momenta

**Figure 3:**
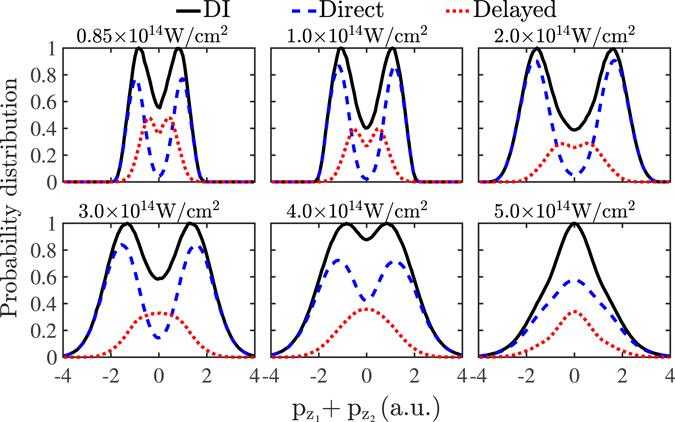
Probability distribution of the sum of the two electron momentum components parallel to the polarization of the laser field (black solid lines) for laser field intensities from 0.85 × 10^14^ W/cm^2^ to 5 × 10^14^ W/cm^2^. For each intensity, the probability distribution of the sum of the momenta of the delayed pathway (red dotted lines) and of the direct pathway (blue dashed lines) are also plotted. The focal volume effect is not accounted for. Each probability distribution is divided by its maximum value.

**Figure 4:**
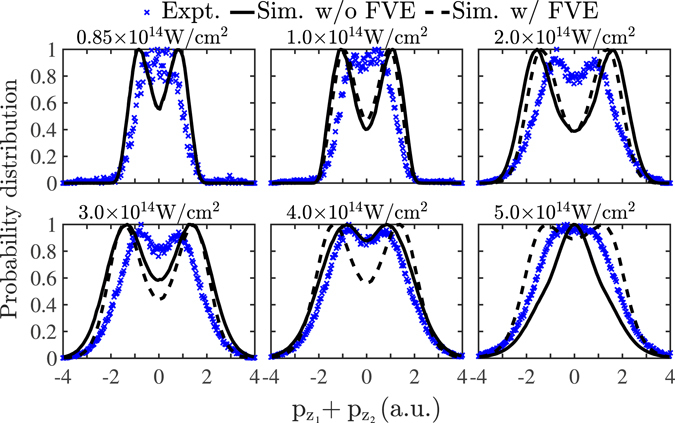
Probability distribution of the sum of the two electron momentum components parallel to the polarization of the laser field for laser field intensities from 0.85 × 10^14^ W/cm^2^ to 5 × 10^14^ W/cm^2^. The computed results with the focal volume effect not accounted for are denoted by black solid lines and when it is accounted for by black dashed lines; the blue crosses denote the experimental results^8^. Each probability distribution is divided by its maximum value.

In Fig. 3, the probability distributions of the sum of the two electron momentum components along the polarization direction of the laser field are presented for intensities from 0.85 × 10^14^ W/cm^2^ to 5 × 10^14^ W/cm^2^. In Fig. 3, the contribution of the direct and the delayed pathways of double ionization to the probability distribution of the sum of the momenta is also shown; the focal volume effect is not accounted for. It is found that the delayed pathway’s contribution is a distribution concentrated around zero while the direct pathway’s contribution is a doubly-peaked distribution, as expected from ref. 10. The direct pathway’s probability distribution of the sum of the momenta is the broadest one. Therefore, including only the delayed pathway of double ionization would result in a narrower distribution of the sum of the momenta than the observed one. Indeed, the 1D model described in ref. 8 which accounts only for the delayed pathway of double ionization results in a narrower probability distribution of the sum of the momenta than the observed one. In Fig. 4, the experimental results for the probability distribution of the sum of the momenta in ref. 8 are compared with one set of computed results that account for the focal volume effect (black dashed lines) and one that does not (black solid lines). It is found that the computed results are in good agreement with the observed ones. Specifically, it is found that, for each intensity, the computed sum of the electron momenta extends over a range that is very similar to the experimental one. For instance, for an intensity of 0.85 × 10^14^ W/cm^2^, the computed sum of the momenta extends over a range from roughly −2 a.u. to 2 a.u., while, for an intensity of 5 × 10^14^ W/cm^2^, it extends from −4 a.u. to 4 a.u.; for both intensities these ranges are in agreement with the experimental results^8^. It is noted that a difference of the computed probability distributions of the sum of the electron momenta with the experimental ones is that the computed ones have smaller values around zero. This is more so the case for the computed results that account for the focal volume effect. This difference suggests that the current 3D model underestimates the contribution of the delayed pathway of double ionization.

### Correlated electron momenta as a function of intensity: transition from strong to soft recollisions

**Figure 5:**
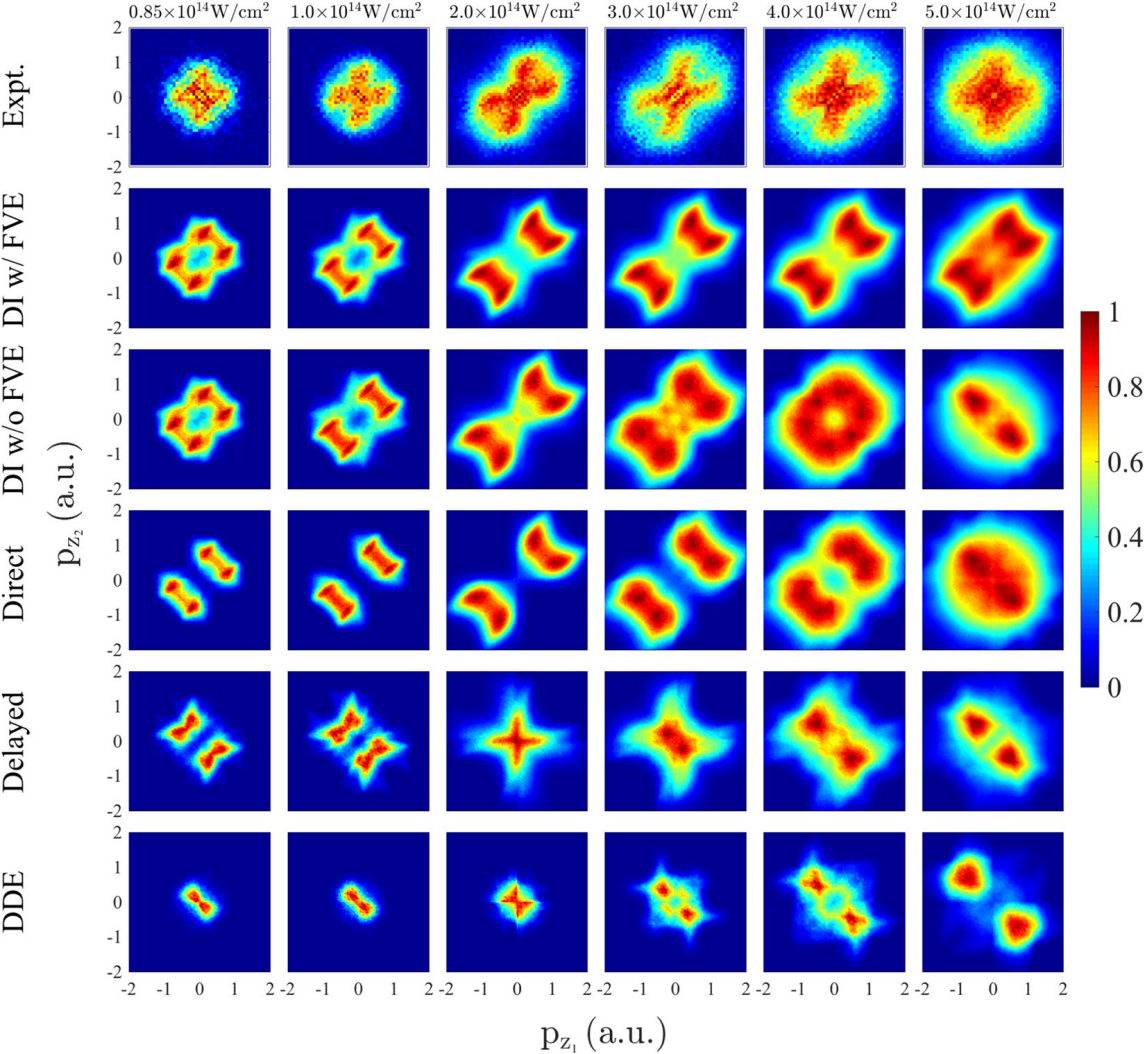
First row: measured correlated electron momenta^8^. Second row: computed correlated electron momenta for all double ionization events with the focal volume effect accounted for. Third row: computed correlated electron momenta for all double ionization events with the focal volume effect not accounted for. Fourth row: correlated electron momenta for the direct pathway of double ionization with the focal volume effect not accounted for. Fifth row: correlated electron momenta for the delayed pathway of double ionization with the focal volume effect not accounted for. Sixth row: correlated electron momenta for the double delayed pathway of double ionization with the focal volume effect not accounted for. The sum of the fourth row plus the fifth row plus the sixth row is equal to the third row. Each double differential distribution is divided by its maximum value.

For intensities ranging from 0.85 × 10^14^ W/cm^2^ to 5 × 10^14^ W/cm^2^, the computed correlated electron momenta with the focal volume effect accounted for are plotted and compared to the measured ones in Fig. 5. We find that at all intensities, but particularly at smaller ones, there are fewer doubly-ionized events with both momenta being close to zero than in the experimentally obtained correlated electron momenta^6, 8^. In Fig. 5, we also plot the computed correlated electron momenta for all doubly-ionized events and for the direct and the delayed double ionization pathways without accounting for the focal volume effect. At intermediate intensities of 2–4 × 10^14^ W/cm^2^, as in the observed correlated electron momenta in ref. 8, the computed correlated electron momenta transition to a well-known pattern^10, 27^. This pattern involves both electrons escaping in the same direction either parallel or antiparallel to the laser field, thus, giving rise to a much higher probability density in the first and third quadrants of the correlated electron momenta, rather than the second and fourth ones. We find that this pattern is due to the direct pathway of double ionization which is the prevailing one at intermediate intensities of 2–4 × 10^14^ W/cm^2^. This pattern is due to strong recollisions where the two electron momentum components along the direction of the laser field are both determined from the vector potential at times just larger than the recollision time. Thus, both electrons escape with similar momenta in the direction along the polarization of the laser field. We also find that at these intermediate intensities the pattern of the correlated electron momenta for the delayed pathway of double ionization is more spread out over all four quadrants and has a significant number of doubly-ionized events with both electron momenta close to zero, as expected from ref. 10.

**Figure 6:**
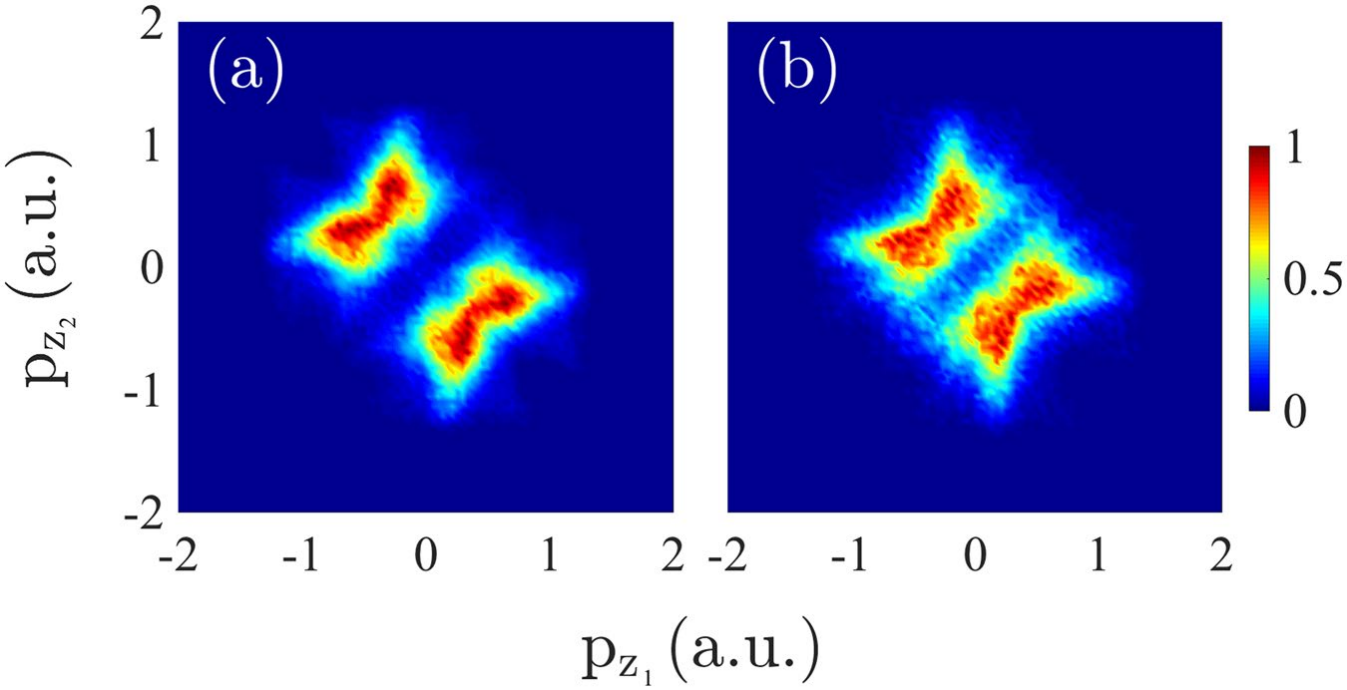
Correlated electron momenta at an intensity of 0.85 × 10^14^ W/cm^2^ for the delayed pathway of double ionization for the case when the electron that ionizes second is electron 2 (**a**) and electron 1 (**b**). Each double differential distribution is divided by its maximum value.

At smaller intensities of 0.85 × 10^14^ W/cm^2^ and 10^14^ W/cm^2^, the computed correlated electron momenta resemble but do not quite have the cross-shaped pattern of the measured results^6, 8^, see Fig. 5. The main difference is that the computed correlated electron momenta have fewer doubly-ionized events with both electron momenta being close to zero. At these small intensities, we find that the direct pathway of double ionization involves mainly events with both electrons escaping in the same direction either parallel or antiparallel to the laser field, however, the electron momenta are not as equal as at intermediate intensities. At small intensities, we also find that the delayed pathway involves mainly doubly-ionized events with both electrons escaping in the opposite direction, with magnitudes of the electron momenta that are more asymmetric than for the direct pathway of double ionization. The cross-shaped pattern is better reproduced by the delayed double ionization pathway, see Fig. 5. Indeed, this pathway does not have as many doubly-ionized events with equal magnitude of the components of the electron momenta along the direction of the laser field as the direct pathway does. In addition, we find that 63% of the events labelled as delayed doubly-ionized satisfy the conditions in eq. 7(b) while 37% satisfy the conditions in eq. 7(a). That is, for the majority of delayed doubly-ionized events the initially bound electron is the one that ionizes last following recollision. We find that the correlated electron momenta when the tunneling electron ionizes second in the delayed doubly-ionized events (37%) resembles more a cross-shaped pattern than the correlated electron momenta when the initially bound electron ionizes second (63%), see Fig. 6.

**Figure 7:**
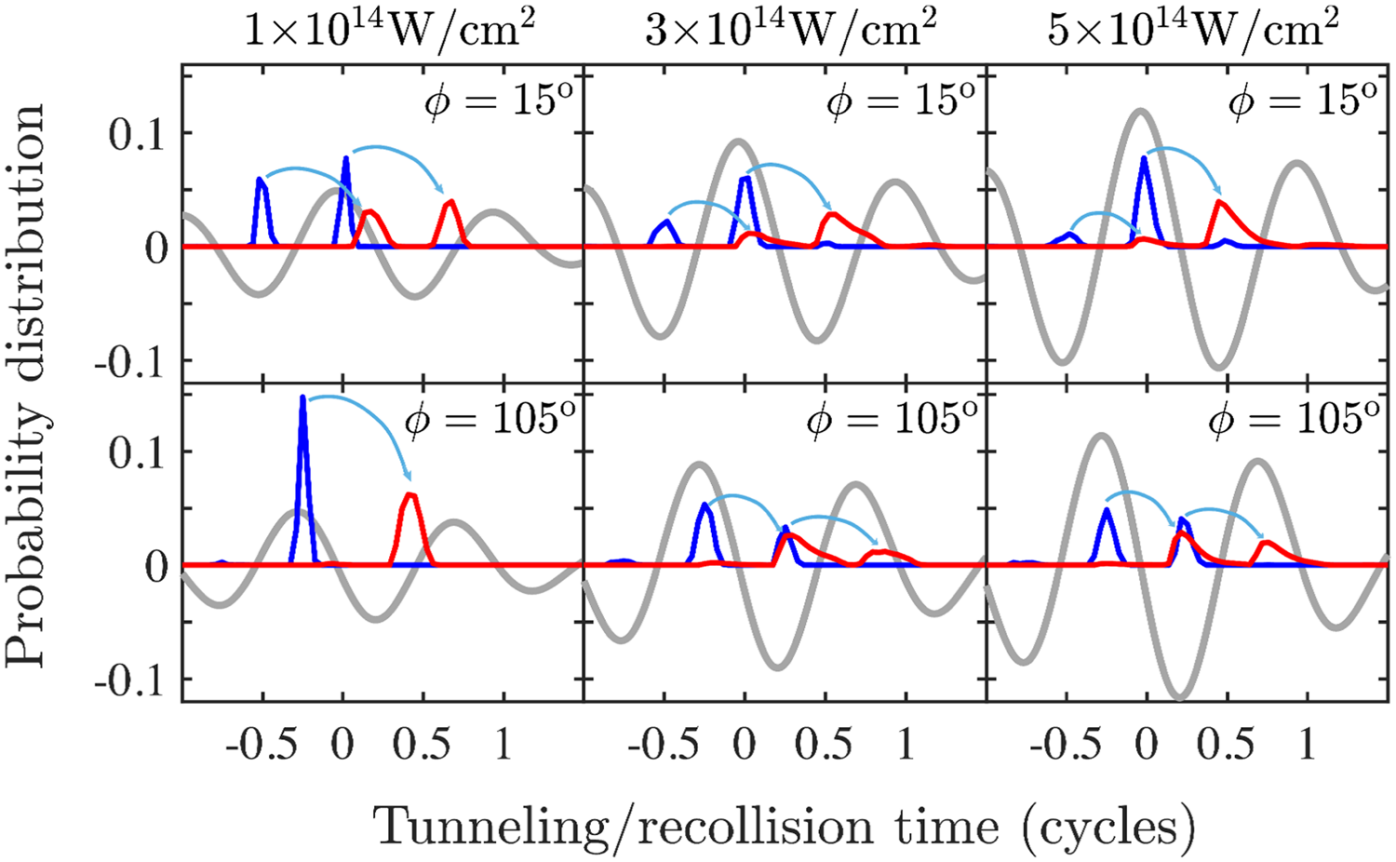
Probability distribution of the tunneling time t_0_ of electron 1 (blue line) and of the recollision time t_rec_ (red line) for intensities 10^14^ W/cm^2^, 3 × 10^14^ W/cm^2^ and 5 × 10^14^ W/cm^2^ and for two different CEPs, *ϕ* = 15° and *ϕ* = 105°, for each intensity. Similar results hold for all other CEPs. Grey line denotes the laser field. The light blue arrows indicate the mapping of a tunneling-time peak to a recollision-time peak.

A less known pattern is that observed experimentally and retrieved computationally with the 3D semiclassical model for intensities above 4 × 10^14^ W/cm^2^, see Fig. 5. For these higher intensities, it is found that the two electrons escape mostly with opposite momenta for a significant number of doubly-ionized events. To identify the reason for this shift in the correlated electron momenta, in Fig. 7, the time electron 1 tunnel-ionizes, t_0_, and the recollision time, t_rec_, are plotted for three different intensities, namely, 10^14^ W/cm^2^, 3 × 10^14^ W/cm^2^ and 5 × 10^14^ W/cm^2^ and for two different CEP’s, namely, *ϕ* = 15° and *ϕ* = 105° for each intensity. The tunneling time of electron 1 is found to be close to the times corresponding to the extrema of the laser field for all three intensities. However, the distribution of the recollision time is found to shift from times corresponding roughly to zeros of the laser field for an intensity of 10^14^ W/cm^2^ to times corresponding to the extrema of the laser field for an intensity of 5 × 10^14^ W/cm^2^. The transfer of energy from electron 1 to electron 2 is much smaller for the soft recollisions. For these higher intensities, where soft recollisions prevail, the momentum of electron 1 is mostly determined from the vector potential at the tunneling time. The momentum of electron 2 is determined by the vector potential shortly after recollision takes place which is roughly half a laser cycle after electron 1 tunnel-ionizes. As a result the two electrons escape mostly with opposite momenta. This mechanism of soft recollisions for higher intensities was first identified in a theoretical study of strongly-driven N_2_ with fixed nuclei^20^. For the delayed pathway of double ionization, this opposite momenta pattern, demonstrated with much higher probability density in the second and fourth quadrants of the correlated electron momenta, sets in at lower intensities of 3 × 10^14^ W/cm^2^, see Fig. 5. For the direct pathway this opposite momenta pattern sets in at higher intensities of 5 × 10^14^ W/cm^2^. This is consistent with a smaller transfer of energy taking place from electron 1 to electron 2 at the recollision time in the delayed pathway compared to the energy transfer in the direct pathway.

### Asymmetry parameter

The asymmetry parameter

10 $${\rm{A}}({\rm{I}},\varphi )=\frac{{{\rm{R}}}_{{\rm{DI}}}^{+}({\rm{I}},\varphi )-{{\rm{R}}}_{{\rm{DI}}}^{-}({\rm{I}},\varphi )}{{{\rm{R}}}_{{\rm{DI}}}^{+}({\rm{I}},\varphi )+{{\rm{R}}}_{{\rm{DI}}}^{-}({\rm{I}},\varphi )}$$

is computed as a function of the intensity I and the CEP (*ϕ*). ${{\rm{R}}}_{{\rm{DI}}}^{+}({\rm{I}},\varphi )$ and ${{\rm{R}}}_{{\rm{DI}}}^{-}({\rm{I}},\varphi )$ denote the percentage of doubly-ionized events with ions escaping with positive and negative momentum, respectively, along the direction of the polarization of the laser field. Since in the 3D semiclassical model the nucleus is fixed, ${{\rm{R}}}_{{\rm{DI}}}^{+}({\rm{I}},\varphi )$ and ${{\rm{R}}}_{{\rm{DI}}}^{-}({\rm{I}},\varphi )$ correspond to the percentage of double ionization events where the sum of the two electrons’ momentum components along the direction of the laser field polarization are negative and positive, respectively. For each intensity, A(I, *ϕ*) is fitted with the sinusoidal function

11 $${\rm{A}}({\rm{I}},\varphi )={{\rm{A}}}_{{\rm{0}}}({\rm{I}})\,sin\,(\varphi +{\varphi }_{{\rm{0}}}({\rm{I}})).$$

**Figure 8:**
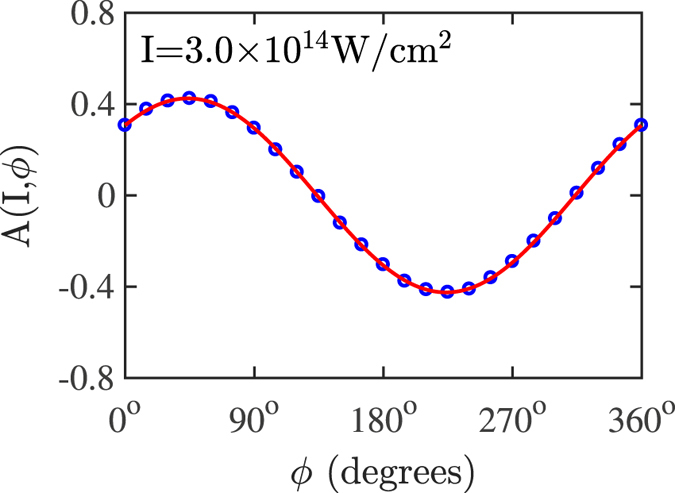
The computed results (blue circles) for the asymmetry parameter A(I, *ϕ*) at I = 3.0 × 10^14^ W/cm^2^ as a function of *ϕ*. The sinusoidal function that is used to fit the computed results is denoted with a red solid line.

In Fig. 8, we illustrate at 3 × 10^14^ W/cm^2^ how the sinusoidal function in Eq. (11) fits our computed results for A(I, *ϕ*) with A_0_ = 0.42 and *ϕ*_0_ = 46°. The computed results show that for a given intensity the percentage of doubly-ionized events with ions escaping with positive versus negative momentum changes as a function of the CEP.

**Figure 9:**
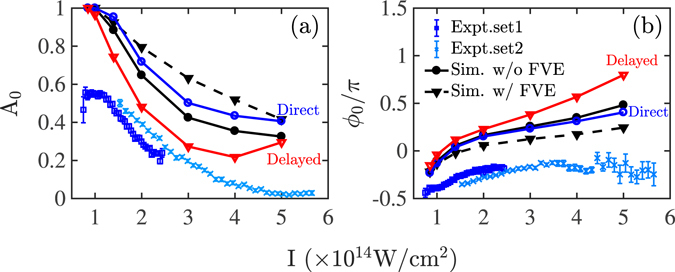
Asymmetry parameters A_0_ (**a**) and *ϕ*_0_ (**b**) as a function of intensity. The computed results for all doubly-ionized events when the focal volume effect is not accounted for are denoted by black solid lines with circles and when it is accounted for by black dashed lines with triangles. The delayed pathway of double ionization is denoted by red solid lines with triangles and the direct pathway by blue solid lines with circles. For the direct and delayed pathways of double ionization the focal volume effect is not accounted for. Experimental results^8^ are denoted by light blue crosses and dark blue squares.

In a manner similar to the one illustrated in Fig. 8, we obtain the computed asymmetry amplitude A_0_(I) and offset phase *ϕ*_0_(I) at other intensities. The computed A_0_(I) and offset phase *ϕ*_0_(I) are plotted in Fig. 9(a,b), respectively, and compared with two sets of experimentally obtained asymmetry parameters^8^. The comparison shows that the 3D semiclassical model reproduces well the decreasing pattern of A_0_ and the increasing pattern of *ϕ*_0_ with increasing intensity. However, the computed values for these asymmetry parameters are higher than the ones obtained from the experimental results. Smaller values of A_0_ correspond to a more spread out pattern of the correlated electron momenta. Thus, the larger values of A_0_ of the computed results are consistent with the computed correlated electron momenta having less doubly-ionized events with sum electron momenta close to zero compared to the measured ones. Moreover, in Fig. 9(a,b) the asymmetry parameters are plotted for each of the main two pathways of NSDI. It is shown that for both pathways the asymmetry parameter *ϕ*_0_(I) has a similar pattern. The asymmetry parameter A_0_(I) for the delayed pathway is generally smaller. This is consistent with the correlated electron momenta of the delayed pathway having a more spread out pattern in all four quadrants than the correlated electron momenta of the direct double ionization pathway, as we have seen in the previous section. Since A_0_(I) is smaller for the delayed pathway of double ionization, the fact that the computed values of A_0_(I) are larger than the measured ones could be due to the fact that the 3D semiclassical model underestimates the contribution of the delayed pathway. We have reached a similar conclusion when discussing the distribution of the sum of the electron momenta.

### Correlated momenta and double ionization pathways as a function of CEP

**Figure 10:**
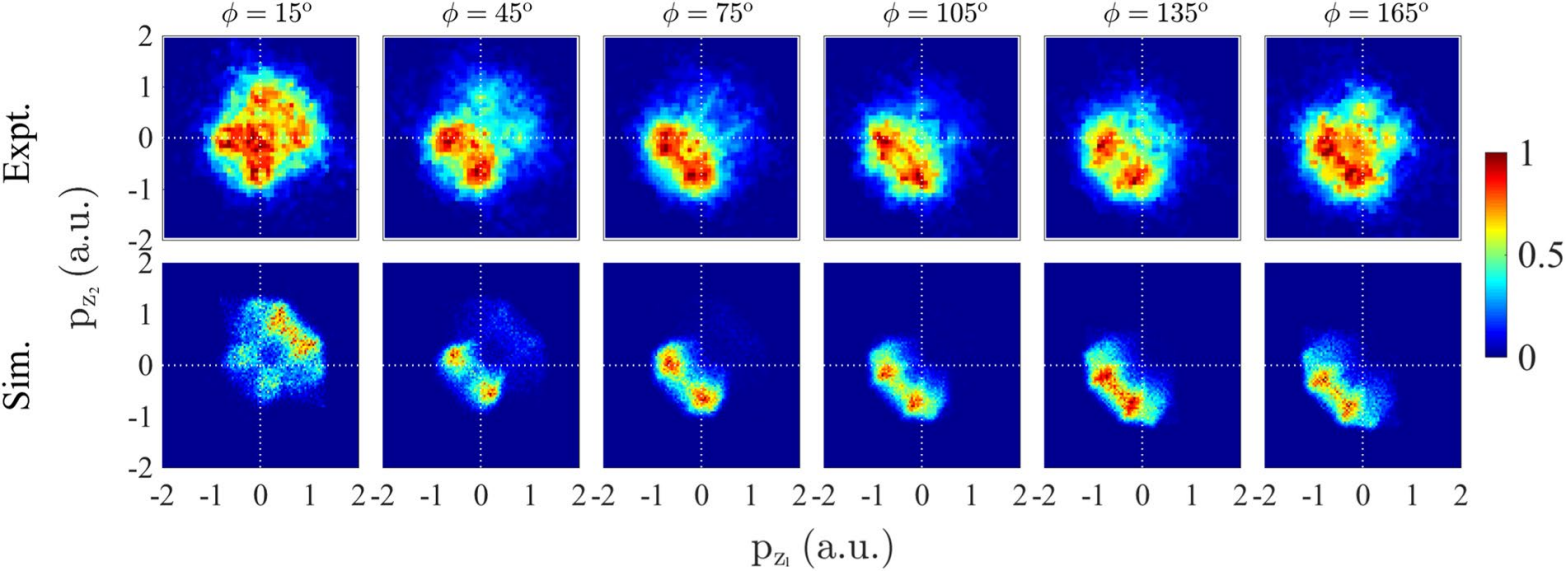
Correlated electron momenta at an intensity of 0.85 × 10^14^ W/cm^2^ for *ϕ* ranging from 15° to 165° with a step of 30°. For *ϕ* ranging from 15° to 165° the experimental results from ref. 28 are compared with the computed results. Each double differential distribution is divided by its maximum value.

In what follows, the dependence of the correlated momenta on the CEP is investigated at an intensity of 0.85 × 10^14^ W/cm^2^. In Fig. 10, the correlated momenta are plotted for *ϕ* ranging from 15° to 165° with a step of 30°. The bin size of the CEP is chosen to be larger than 200 mrad, which is the experimental precision of the CEP, and large enough in order to get good statistics. For data analysis, all events are selected for which one electron has been detected in coincidence with an Ar^2+^ ion. The momentum of the second electron, which is not detected for most events, is calculated from conservation of momentum. Both the data and the computed results are symmetrized with respect to the bottom-left-to-top-right diagonal in order to account for the two electrons being indistinguishable. Moreover, due to the symmetry of the Hamiltonian, when *ϕ* → *ϕ* + 180° then **p** → −**p**. This symmetry is respected by the computed results. In the experimental results there is a small deviation from this symmetry. This deviation arises from artifacts of the electron spectrometer and false coincidences. For each CEP, in the top right half of the correlated electron momenta plot the impact of false coincidences is stronger than in the bottom left half. For CEP ranging from 195° to 345° the correlated electron momenta plots have more doubly-ionized events in the top right half. Thus, in Fig. 10, we compare the computed results with the measured correlated electron momenta which are more accurate, i.e. for CEP ranging from 15° to 165° where the correlated electron momenta have more doubly-ionized events in the bottom left half.

**Figure 11:**
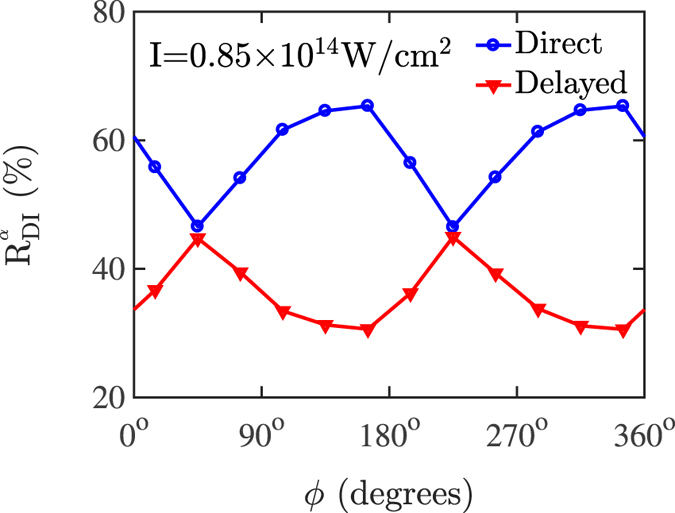
Percentage contribution of the direct and the delayed pathways of double ionization as a function of the CEP at an intensity of 0.85 × 10^14^ W/cm^2^.

A good agreement is found between the computed and the experimental results for CEP ranging from 15° to 165° given the experimental uncertainty of 200 mrad in the CEP. Specifically, the computed correlated momenta correctly reproduce the overall observed pattern for each individual CEP. A difference between the computed and the experimental results is that the former results have less doubly-ionized events with both electron momenta close to zero suggesting that the computations overestimate the contribution of the direct pathway. To better illustrate the change of the correlated momenta pattern as a function of the CEP plotted in Fig. 10, in Fig. 11 the percentage of the direct and delayed pathways of double ionization are plotted as a function of the CEP. At an intensity of 0.85 × 10^14^ W/cm^2^ the delayed pathway has the largest contribution for CEPs *ϕ* = 45° and *ϕ* = 225°, while it has the smallest for CEPs *ϕ* = 165° and *ϕ* = 345°. In Fig. 10, a comparison of the correlated momenta between *ϕ* = 45° and *ϕ* = 165° shows that there is a higher probability density for both electrons to ionize with the same large momentum, with both electrons escaping in the direction that is opposite to the electric field, for *ϕ* = 165° than for *ϕ* = 45°. This is indeed consistent with the direct ionization pathway having a larger contribution for *ϕ* = 165° than for *ϕ* = 45° as shown in Fig. 11. From Fig. 11, it is found that the contribution of each of the two main pathways of double ionization varies roughly by 20% as a function of the CEP for the smallest intensity of 0.85 × 10^14^ W/cm^2^.

## Conclusions

Using a 3D semiclassical model we investigate the dependence of double ionization observables on the intensity and on the carrier envelope phase of a near-single-cycle near-infrared laser field employed to drive Ar. The good agreement of the computed results with recent experiments employing near-single-cycle laser pulses^6, 8, 28^, adds to previous successes of this 3D model in identifying features of non-sequential double ionization of two-electron atoms when driven by many-cycle laser pulses^11, 15, 19^. A difference between the computed and the experimental results was found to be a lower value of the distribution of the sum of the two electron momentum components along the direction of the polarization of the laser field and of the correlated electron momenta around zero. This seems to suggest that the current 3D model overestimates the Coulomb attraction of each electron from the nucleus. Future studies can improve on the 3D model for many electron atoms such as Ar by using more accurate effective potentials for the time propagation. Moreover, it was demonstrated that the main pathways of double ionization, that is, the direct and the delayed pathways, both significantly contribute at all intensities currently under consideration. Furthermore, the prevalence of the direct versus the delayed pathway was investigated as a function of the CEP for an intensity of 0.85 × 10^14^ W/cm^2^ and it was shown that the results obtained are consistent with features of the observed correlated electron momenta^28^. Finally, a previously-predicted in the context of a strongly-driven fixed-nuclei N_2_ unexpected anti-correlation momentum pattern at higher intensities^20^, is observed experimentally in the context of strongly-driven Ar^8^ and also reproduced in the current work for strongly-driven Ar by a near-single-cycle laser field. It is shown that this anti-correlation pattern is due to soft recollisions with recollision times close to the extrema of the laser field.
